# Health promotion programs related to the Athens 2004 Olympic and Para Olympic games

**DOI:** 10.1186/1471-2458-6-47

**Published:** 2006-02-24

**Authors:** Elpidoforos S Soteriades, Christos Hadjichristodoulou, Jeni Kremastinou, Fotini C Chelvatzoglou, Panagiotis S Minogiannis, Matthew E Falagas

**Affiliations:** 1 Alfa Institute of Biomedical Sciences (AIBS), Athens, Greece; 2 Cyprus International Institute for the Environment and Public Health in Association with Harvard School of Public Health, Nicosia, Cyprus; 3 Department of Hygiene and Epidemiology, Medical Faculty, University of Thessaly, Larisa, Greece; 4 National School of Public Health – Olympic Planning Unit (OPU), Athens, Greece; 5 Department of Medicine, "Henry Dunant" Hospital, Athens, Greece; 6 Health Services, Athens 2004 Olympic Games Organizing Committee, Greece; 7 Department of Medicine, Tufts University School of Medicine, Boston, Massachusetts, USA

## Abstract

**Background:**

The Olympic Games constitute a first-class opportunity to promote athleticism and health messages. Little is known, however on the impact of Olympic Games on the development of health-promotion programs for the general population.

Our objective was to identify and describe the population-based health-promotion programs implemented in relation to the Athens 2004 Olympic and Para Olympic Games.

**Methods:**

A cross-sectional survey of all stakeholders of the Games, including the Athens 2004 Organizing Committee, all ministries of the Greek government, the National School of Public Health, all municipalities hosting Olympic events and all official private sponsors of the Games, was conducted after the conclusion of the Games.

**Results:**

A total of 44 agencies were surveyed, 40 responded (91%), and ten (10) health-promotion programs were identified. Two programs were implemented by the Athens 2004 Organizing Committee, 2 from the Greek ministries, 2 from the National School of Public Health, 1 from municipalities, and 3 from official private sponsors of the Games. The total cost of the programs was estimated at 943,000 Euros; a relatively small fraction (0.08%) of the overall cost of the Games.

**Conclusion:**

Greece has made a small, however, significant step forward, on health promotion, in the context of the Olympic Games. The International Olympic Committee and the future hosting countries, including China, are encouraged to elaborate on this idea and offer the world a promising future for public health.

## Background

International sporting competitions in general, and the Olympic Games in particular, represent important events, where elite athletes and millions of people from around the world gather together to celebrate athleticism[1,2]. At the same time, mass media broadcasting of the Olympic Games to billions of people worldwide constitute a powerful tool through which messages on athletic values and health promoting habits and lifestyles may be presented[3-5]. Furthermore, the city hosting the Olympic Games is frequented by a large number of visitors from different geographic areas, and provides an ideal setting for health promotion for the visitors as well as the city's local population[6-8]. Thus, international sport events represent a great opportunity for public health professionals to promote healthy messages, though many companies, e.g. the tobacco industry, also take the opportunity to advertise unhealthy products at certain sport events[9-14].

Most international sponsorship activities have involved several strategies for health promotion including the promotion of health messages,[15,16] the use of role models,[17] the development of policies that support health behaviors such as the designation of smoke-free areas,[18,19] and physical activity,[20] and the restrictions on tobacco or alcohol sponsorship or cigarette sale[21,22]. In addition to sponsorship activities during sport events, a range of non-sponsorship activities have also been associated with the Olympic Games[23]. Since the first smoke-free Calgary Winter Olympics in 1988, the International Olympic Committee (IOC) has refused sponsorship and publicity from the tobacco industry and all subsequent Games have been labeled smoke-free[24].

Over the past decades, great efforts have been made in identifying and promoting healthy habits in the general population, particularly through sports. [25-27] Smoking, lack of physical activity, and poor diet constitute a host of modifiable risk behaviors that are targeted by public health professionals. Similarly, obesity represents an additional target for public health programs as is dependent on the above unhealthy behaviors. The Olympic Games provide a first-class opportunity to promote healthy messages to the general population and inspire people of all ages to become fit, and improve their health.

The purpose of our study was to evaluate the impact of the Athens 2004 Olympic and Para Olympic Games on the development and implementation of population-based health-promotion programs and related activities for the benefit of the spectators and the local population.

## Methods

A cross-sectional survey was conducted in order to collect information from all potential stakeholders in Greece regarding health promotion programs developed and implemented in relation to the Athens 2004 Olympic and Para Olympic Games. The Athens 2004 Organizing Committee, all ministries of the Greek government, the National School of Public Health, all municipalities that hosted Olympic events and all official private sponsors of the Games were invited to participate in the survey.

Two questionnaires were developed in order to obtain information about health-promotion programs associated with the Olympic and Para Olympic Games one for the sponsors and one for the ministries involved in the organization of the games [see additional files 1 and 2]. Information was collected regarding the type of health promotion program implemented, the population targeted, the cost of each program and the time period covered by each program. In particular, information on physical activity, anti-smoking campaigns, diet, and alcohol consumption was specifically requested. The survey included additional open-ended questions in order to identify other programs that might have been implemented. The survey focused on a time period extending from up to two years prior to the Olympic Games (2002, 2003), until the period of the Olympic Games itself (August 13^th ^– September 30th, 2004).

The questionnaire was mailed or faxed to each responsible officer from all above-described agencies, in order for them to review it and gather information with regards to the questions of the survey, in advance of the data collection process. After the distribution of the questionnaire, an investigator contacted each officer from all agencies and completed each survey over the phone or requested a completed survey via facsimile. Repeated phone calls to each responsible officer were placed until an official reply to our request or a non-cooperation statement was provided.

The data collection process was completed four months after the conclusion of the Games. Additional analytic data on the cost of the Games in general and on specific components of the Games in particular were obtained from the Ministry of National Economy.

## Results

A total of 44 agencies were contacted by phone and/or facsimile, 40 agencies completed the questionnaire, and 10 health-promotion programs, implemented prior to, and/or during the Olympic Games, were identified. The average cost for the implemented programs was about 95,000 Euros (€) with a range between 20,000€ and 600,000€. The total cost of the health-promotion programs was about 943,000€, and constituted a small fraction (0.08%) of the overall cost of the games, even after excluding the cost of construction, preparation of athletic facilities, and security.

The Athens 2004 Olympic Games Organizing Committee implemented two programs: (a) a non-smoking policy in all premises related to the Organizing Committee and (b) the distribution of condoms, free of charge, in the Olympic Village Polyclinic. In the Table, we describe the different programs implemented along with the responsible agency, the type of activity, the time period covered and the target population. The vast majority of the programs included the development and distribution of brochures on several health-related topics targeting visitors, spectators of the Games, and the general population.

## Discussion

Greece is one of the financially weakest and demographically smallest countries ever managed to organize the Olympic Games in a quite successful way. Despite significant shortage of funds and financial strain on the country's budget, several agencies devoted some resources on health-promotion programs implemented prior to and during the Olympic Games for the benefit of visitors, spectators, and the general population. While the cost of the implemented programs was limited compared to the total expenses of the Games, we believe that it offers a hopeful seed for the future.

There are limited reports in the medical literature on the development and implementation of health-promotion programs related to sporting events in general [10,11,18,20,22,26] and the Olympic Games in particular[23,28]. Many competitions take place all over the world, however the benefit to each hosting country's population remains unclear[29-31]. Although more programs could have been developed, the Athens 2004 Olympic Games offered a small, nevertheless, important example to be expanded and build upon in the future, in order to diffuse health messages, and encourage healthy habits in the general population in the period leading to the Games, as well as during the Olympic Games[32].

Developing effective and sustainable health promotion programs in relation to the Olympic Games remains an important challenge for public health professionals around the world. Each country undertakes significant economic investments to organize the Olympic Games. Therefore it would be instrumental to include health promotion programs as a high priority in the overall strategic planning for the Games. Each organizing country along with the International Olympic Committee could work together in developing population-based programs such as competitions for the lay public, promotion of healthy life styles through athletes acting as role models, development of exercise fields and stadiums in underdeveloped areas etc. Perhaps such health promotion programs with appropriate scoring criteria should be incorporated into the application and evaluation process of candidate cities and countries for the prospective organization of Olympic Games.

Our study was limited in scope and despite obtaining information in a cross-sectional fashion our data collection covered a period of two years in a retrospective fashion. Although, a large number of public and private agencies were surveyed, it is possible that we might have missed some information, which could have resulted in underestimation of implemented health-promotion programs. In addition, information on the cost of the different programs may not reflect accurate economic reports since we relied on the information reported in the questionnaire.

Our findings suggest that there is significant room for improvement in the development and implementation of health-promotion programs during sport events. The organization of the Olympic Games, constitute an excellent opportunity for public health professionals to promote health in the general population of the hosting country and distribute such messages around the world. "Whenever there is a will there's a way" as evidenced by the institutional policies developed by the International Olympic Committee regarding doping and smoke-free areas[33-36]. Greece has made a small, however, significant step forward on health promotion in relation to the Olympic Games. The International Olympic Committee and China are encouraged and expected to elaborate on this idea and offer the world a promising future for public health.

## Competing interests

The author(s) declare that they have no competing interests.

## Authors' contributions

ESS conceived of the study, and participated in its design and coordination. ESS, CH, JK, and MEF developed the questionnaires. ESS, CH, JK, FCC, and PSM collected the data. ESS drafted the manuscript. All authors made contributions to the text and read and approved the final manuscript.

## Pre-publication history

The pre-publication history for this paper can be accessed here:



## Supplementary Material

Additional File 1 Questionnaire on Health Promotion Programs Related to the Athens 2004 OlympicGames (Ministries, Government Agencies) Click here for file

Additional File 2 Questionnaire on Health Promotion Programs Related to the Athens 2004 OlympicGames (Sponsors) Click here for file

## Figures and Tables

**Table 1:** Health promotion programs implemented prior to and/or during the Athens 2004 Olympic and Para Olympic Games

**Agency**	**Number of Programs**	**Type of Activity (cost)**	**Time Period**	**Target Population**
Athens 2004 Organizing Committee	Two (2)	1. Non-smoking policy (€25,000) 2. Free distribution of condoms (€25,000)	1. For two years prior to, and during the Games 2. During the Games	1. Spectators, volunteers, and staff of AOC 2. Everyone who had access to the Olympic village
Ministries of the Greek Government	Two (2)	1. Brochures and CD- ROM (three languages) to promote the Mediterranean diet. Exhibit of traditional food products (€28,000) 2. Brochure with travel-related health information about Greece (€20,000)	1. For six months prior to the Olympic Games and during the Olympic period 2. During the OlympicPeriod	1. Journalists and the general population 2. Visitors of Greece
The National School of Public Health	Two (2)	1. Brochure/Posters on how to prevent heat disorders (€10,000) 2. Brochure for healthy swimming (€15,000)	1. For three months prior to, and during the Olympic period 2. During the Olympic Period	1. Athletes, visitors and the general population 2. Visitors and the general population
Municipalities Hosting Olympic Events	One (1)	1. Brochure on healthy cities (€60,000)	1. For two years prior to the Games	1. The general population
Official Private Sponsors of the Games	Three (3)	1. Brochures and TV/Radio advertisements on physical activity (N/A) 2. Brochures, TV/Radio advertisements, and lectures on physical activity and the Olympic Games (€600,000) 3. Brochure on diet (N/A)	1. For two years prior to, and during the Games 2. For two years prior to, and during the Games 3. For one year prior to the Games	1. The general population, spectators, students, and women 2. School students 3. School students
