# Supplementary material for: Health promotion programs related to the Athens 2004 Olympic and Para Olympic games
Source: BMC Public Health. 2006 Feb 24;6:47. doi: 10.1186/1471-2458-6-47 (PMC1397814; doi:10.1186/1471-2458-6-47)
Supplement: Additional File 2 — Questionnaire on Health Promotion Programs Related to the Athens 2004 OlympicGames (Sponsors) [file 1471-2458-6-47-S2.doc]

**Questionnaire on Health Promotion Programs**

**Related to the Athens 2004 Olympic Games**

**(Sponsors)**

1. Have you ever developed and implemented health promotion programs regarding the Olympic Games at schools, other institutions or certain population groups?
   1. Yes
   2. No
2. What type of program was implemented?
   1. Physical activity (exercise)
   2. Antismoking campaign
   3. Healthy Diet
   4. Alcohol consumption
   5. Other: ……………………………………………………….

3. What was the target population?

1. General population
2. Spectators of the games
3. Students
4. Elderly
5. Women
6. Solders
7. Other: …………………………………………………………….
8. What method has been used for the implementation of the program?
   - 1. Campaign with written materials (posters, brochures)
     2. Mass media campaign (T.V., radio, newspapers, magazines)
     3. Lectures offered to the target population
     4. Events with active participation of the target population
     5. Other: ……………………………………………………………..
9. What were the means used in the program?
   - 1. Written materials (brochures, posters)
     2. Written media (newspapers, magazines)
     3. Radio
     4. Television
     5. Other: ……………………………………………………………..
10. What was the total cost of the program?
    1. …………………………………………
11. When was the program implemented?
    - 1. Before the Olympic Games
      2. Before and during the Olympic Games
      3. Only during the Olympic Games
      4. Other:………………………………..
12. If the program was implemented prior to the Olympic Games, what was the duration of its implementation?
    - 1. 2 years before the Olympic Games
      2. 1 year before the Olympic Games
      3. 6 months before the Olympic Games
      4. 3 months before the Olympic Games
      5. Other: ………………………………………….
